# Analysis of the relationship between EEG burst suppression and poor prognosis in children under general anaesthesia: study protocol for a prospective, observational, single-centre study

**DOI:** 10.1186/s13063-023-07478-8

**Published:** 2023-07-28

**Authors:** Qian Xu, Jianmin Zhang, Zhengzheng Gao, Shanshan Li, Gan Li

**Affiliations:** Department of Anesthesiology, National Center for Children’s Health, Beijing Children’s Hospital, Capital Medical University, No.56, South Lishi Road, Beijing, 100045 China

**Keywords:** Emergence delirium, Burst suppression, EEG, General anaesthesia

## Abstract

**Background:**

Emergence delirium (ED) in children refers to the immediate postoperative period when children experience decreased perception of their surroundings, accompanied by disorientation and altered perception. Burst suppression (BS) is recognised as periods longer than 0.50 s during which the EEG does not exceed approximately + 5.0 mV, which is an electroencephalographic state associated with profound inactivation of the brain. Our primary objective was to determine the association between BS on electroencephalogram (EEG) under general anaesthesia with postoperative wake-up delirium and multiple adverse outcomes, such as prolonged awakening and extubation.

**Methods:**

In this prospective, observational cohort study at Beijing Children’s Hospital, Capital Medical University, Beijing, China, children aged 6 months to 9 years who underwent surgery under general anaesthesia and underwent EEG monitoring between January 2022 and January 2023 were included. Patients’ prefrontal EEGs were recorded intraoperatively as well as analysed for the occurrence and duration of BS and scored postoperatively for delirium by the PAED scale, with a score of no less than 10 considered as having developed wake-up delirium.

**Discussion:**

This study identified a relationship between EEG BS and postoperative awakening delirium under general anaesthesia in children and provides a novel preventive strategy for postoperative awakening delirium and multiple adverse outcomes in paediatric patients.

**Trial registration:**

Chinese Clinical Trial Registry, ChiCTR2200055256. Registered on January 5, 2022.

## Background

Paediatric awakening delirium often presents in the PACU (anaesthesia recovery room) and is characterised as acute brain dysfunction in the awakening phase after general anaesthesia, accompanied by disorganised thinking and loss of directional force. The condition is often associated with factors such as age, anaesthesia modality, and postoperative pain. Burst suppression is an electroencephalographic model in which high amplitude sharp waves alternate with inhibitory electroencephalogram (EEG) activity. Intraoperative burst suppression is indicative of an excessively deep state of anaesthesia. Furthermore, studies have shown that during general anaesthesia surgery, intraoperative burst suppression is associated with the presence of postoperative delirium [1, 2]. A period of burst suppression and low index level anaesthesia may not only cause postoperative delirium, but the incidence of postoperative delirium increases with an increase in the duration of intraoperative EEG suppression [1, 3]. Moreover, in elderly patients, the incidence of delirium significantly increases with age > 65 years [4].

Sevoflurane is an inhaled general anaesthetic drug that is primarily used in the induction and maintenance of paediatric clinical anaesthesia and has the advantages of high efficiency, stationarity, and easy control. Sevoflurane has a low blood/gas partition coefficient, and maintaining anaesthesia with these agents increases the risk of emergence delirium (ED). The incidence of ED in children receiving sevoflurane anaesthesia has been reported to be as high as 80%, much higher than that in adults [5]. Propofol is an intravenous anaesthetic drug that is widely used in clinical practice and has a rapid onset of action, rapid recovery, and reduced incidence of postoperative nausea and vomiting. It binds via the postsynaptic γ-aminobutyric acid type A (GABAA) receptors, which induce an inward chloride current that hyperpolarizes the postsynaptic neuron, thus leading to inhibition, an effect that favours the appearance of slow δ oscillations. Chandler [6] et al. found a lower incidence of ED when using propofol remifentanil for total intravenous anaesthesia (TIVA) compared with the use of sevoflurane anaesthesia. In studies of children, burst suppression appeared easily during the induction phase of general anaesthesia, and the younger the child’s age, the greater the number of burst suppression appearances. It has been demonstrated that the slowing of intraoperative EEG correlates with burst suppression [7]; however, studies focusing on the intraoperative burst suppression period and the correlation with ED in children remain scarce. Therefore, it is necessary to investigate whether the occurrence and duration of burst suppression patterns on EEG during general anaesthesia in children are associated with the occurrence of ED.

## Methods

### Study design

This study was a prospective, observational, single-centre study. This study was approved by the ethics committee of Beijing Children’s Hospital (IEC-C-006-A04-V.06) and registered in the Chinese clinical trial registry (CHICTR2200055256). We strictly adhered to clinical practice guidelines and the Declaration of Helsinki throughout the trial. The study consisted of patients aged 0.5–9 years for whom surgery requiring general anaesthesia was planned, and the length of surgery was greater than 30 min. Informed consent was obtained by communicating with the parents or guardians of eligible participants prior to the onset of study operations, and written informed consent was obtained from the parents or guardians of potential study participants.

Using SedLine EEG monitors (SedLine, Masimo Inc, Irvine, CA, USA) with a single-use sensor on the forehead, four channels were recorded corresponding to the international nomenclature of EEG for FP1 FZ, FP2 FZ, F7 FZ, and F8 FZ. EEG recordings were started before induction of anaesthesia and ended after extubation or removal of the laryngeal mask. EEG recordings were continued until the patient was fully awake. EEG analysis focused on the incidence of intraoperative burst suppression events, which was assessed by visual inspection of the original EEG when the patient was under deep anaesthesia, at which point EEG activity entered a state of suppression with alternating high-voltage and flat states, eventually progressing to a sustained flat phase, which we called an isoelectric event. If the duration of the isoelectric line in the burst suppression segment exceeded 0.5 s, then burst suppression was determined to have occurred. The duration of intraoperative burst suppression was calculated from the first isoelectric segment to the end of the last isoelectric line. ED was assessed according to PAED scale scores, which were scored from the end of the procedure by the same research team member (anaesthesiologists or nurse anaesthetists) using the paediatric anaesthesia awakening delirium score within 15 min of extubation and within 15–30 min of extubation. The primary outcome was the peak PAED score achieved in the postoperative recovery room, with wake-up delirium defined as a PAED score of ≥ 10.

### Research purpose

The main purpose of this study was to determine the relationship between the suppression of EEG bursts under general anaesthesia in children and postoperative delirium and various adverse prognoses, such as prolonged awakening time and prolonged extubation time. The secondary purpose was to understand the incidence of EEG burst suppression in children during surgery and analyse the relevant influencing factors (participants, surgery, anaesthesia factors, etc.) under the current clinical anaesthesia methods in Beijing Children’s Hospital.

### Sample size

**Fig. 1:**
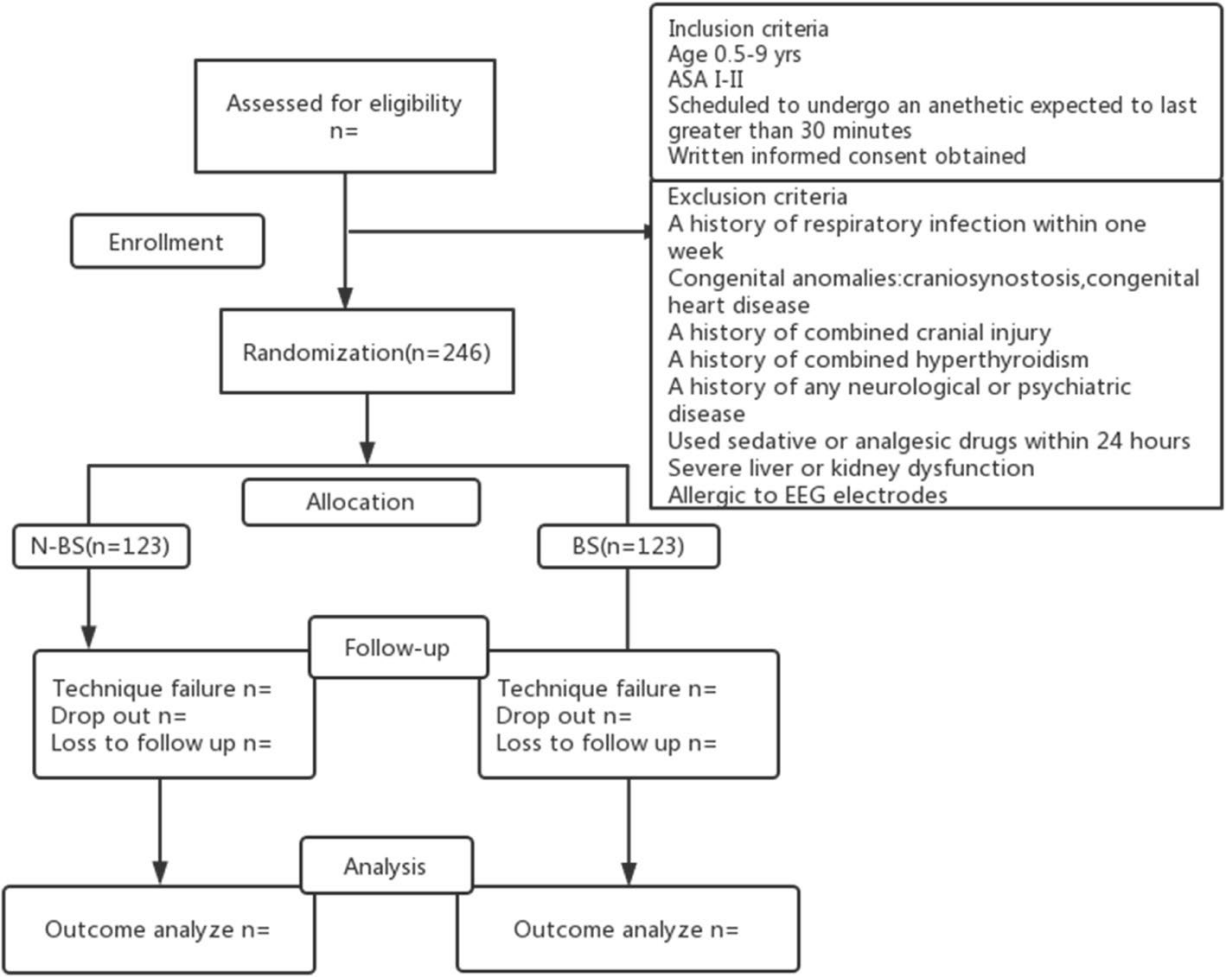
Participant flow

According to the pre-experimental results, the incidence of delirium during awakening in the group with burst suppression during operation was 47%, and the incidence of delirium during awakening in the group without explosive suppression during operation was 28%, which was calculated using the tests for a two aspects design model in PASS, *α* = 0.05, *β* = 0.8, two-sided, 1:1 group allocation. The result was that each group needed 98 participants, and the estimated data loss rate was 20%. The final sample size was 123 participants for each group or 246 participants for both groups. If the actual shedding rate is greater than 20%, in order to ensure sufficient sample size, the study duration will be prolonged beyond the anticipated end date. A flow chart of the research design is shown in Fig. 1.

### Research object

#### Inclusion criteria

1. American Association of Anesthesia (ASA)I-II
2. Age: 6 months–9 years old (children’s EEG changes are most obvious at 0–3 years old [8, 9], and children’s delirium most often occurs at 4–9 years old [10]), regardless of sex)
3. The duration of general anaesthesia operation was more than 30 min
4. Knowledge of the purpose and content of this study and written informed consent

#### Exclusion criteria

1. Participants with a history of respiratory tract infection within 1 week
2. Participants with congenital malformations such as craniocerebral malformation and congenital heart disease
3. Participants with craniocerebral injury, possible intracranial hypertension, cerebral aneurysm, cerebrovascular accident, and central nervous system disease
4. Participants with a personal and/or family history of hyperthyroidism
5. Participants with a personal and/or family history of neurological or psychiatric diseases
6. Participants who used sedative or analgesic drugs within 24 h
7. Participants with contraindications to general anaesthesia
8. Participants with severe liver and kidney dysfunction
9. Participants who had an allergy or adverse reactions to EEG electrodes

### Study duration and safety

This study lasted for 1 year from January 2022 to January 2023.

The patients’ families could choose to withdraw from the study at any time without losing any benefits. Considering the safety of patients, it was possible to conduct a relevant examination after withdrawal.

### Violation of the definition of the study

If the patient did not have at least 30 min of EEG record and interpretable EEG waveform during general anaesthesia, it was considered a violation of this study.

### Definition of research objectives

#### Main research objectives

The main research objective is to determine the relationship between EEG burst suppression under general anaesthesia and postoperative delirium and various adverse prognoses in children.

The following five values were used to describe the occurrence of burst suppression:

1. Occurrence rate of burst suppression events
2. Total number of burst suppression events
3. Total time of occurrence of burst suppression event
4. Average duration of each burst suppression event
5. Percentage of total burst suppression occurrence time to total anaesthesia time

#### Secondary research objectives

The secondary objectives were to determine the perioperative factors related to the occurrence of burst suppression events, which included patient factors, anaesthesia factors, surgical factors, and physiological factors (see the “Discussion” section) and to determine whether the incidence of burst suppression is related to the emergence of delirium during the patient’s waking period.

### Definition of study period

Five time points of anaesthesia induction, intubation/laryngeal mask, skin cutting, stopping anaesthesia and maintaining drugs, and extubation were recorded. These five time points formed four anaesthesia stages: (1) induction stage—anaesthesia induction to completion of intubation or laryngeal mask insertion; (2) preoperative stage—from the insertion of intubation or laryngeal mask to the beginning of surgery; (3) anaesthesia maintenance stage—skin cutting to maintenance drug stop; (4) resuscitation stage—anaesthesia maintenance drug stops until tracheal intubation or laryngeal mask was removed.

### Research and analysis variables

#### Patient factors

- Sex (male/female)
- Age at the time of study (months)
- Premature birth: birth at < 37 weeks (yes/no)
- Height at the time of study (cm)
- Body weight at the time of the study (kg)
- Duration of fasting water intake during the operation (h)
- ASA physical condition (1, 2)
- Anaesthesia-induced anxiety state: PAB score

#### Anaesthesia factors

- Anaesthesia induction mode: intravenous or inhalation induction
- Application of nerve block (yes/no)
- Intubation method: laryngeal mask insertion or endotracheal intubation
- Anaesthesia maintenance mode: intravenous anaesthesia or inhalation anaesthesia, dexmedetomidine (yes/no)
- Application of other analgesics to assist analgesia: ibuprofen (yes/no), ketamine (yes/no), nalbuphine (yes/no), and pentazocine (yes/no)
- Anaesthesia duration (min)
- Face, Legs, Activity, Cry, and Consistency Scale (FLACC): the highest score (0–10) within 15 min after tube removal
- Richmond agitation scale score (− 5–4) within 15 min after extubation
- Relief measures of analgesia/sedation given during recovery (yes/no)
- Peak value of the PAED scale score (0–20) within 15 min after extubation
- Face, Legs, Activity, Cry, and Consistency Scale (FLACC): the highest score (0–10) within 15–30 min after tube removal
- Richmond agitation scale score (− 5–4) within 15–30 min after extubation
- Peak value of PAED scale score (0–20) at 15–30 min after extubation

#### Operation factors

- Operation type: urology, general surgery, orthopaedics, newborn, chest, burn plastic surgery, ophthalmology

#### Physiological factors

At each stage of anaesthesia, the average of the following observations was calculated for each patient:

- SpO_2_(%)
- SBP (mmHg)
- MAP (mmHg)
- Heart rate (beats/minute)
- Temperature (°C)
- End-expiratory carbon dioxide partial pressure (mmHg)
- Airway resistance (cmH2O)

### Processing missing data and research bias

In this study, there were different sources of potential missing data. If not all baseline vital signs were collected, data loss may have occurred before anaesthesia induction. During the operation, if vital signs (MAP, heart rate, etc.) were not fully recorded, data would also be missing. For example, when a blood pressure cuff circulates for several minutes, the blood pressure can be recorded, and the temperature data of the child is incomplete because the temperature is not connected in time. To assess the risk of bias due to absence, we conducted a sensitivity analysis to determine whether the patients without complete records were different from the patients with complete records in terms of demographic characteristics or medical conditions.

### Observation indicators

The main outcome measures were:

- To determine the incidence of sudden depression and delirium in the recovery period of children in this cohort when undergoing general anaesthesia surgery
- Burst suppression: specific results were obtained from offline analysis of the SedLine EEG monitor
- Delirium in awakening period: The same research team member used the paediatric anaesthesia delirium score (Table 1, Paediatric Anaesthesia Emergence Delirium scale, PAED) and the face, leg, activity, crying, and comfortability score (Table 2, Face, Legs, Activity, Cry and Consistency scale, FLACC) to score delirium and pain repeatedly within 15 min and 15–30 min after extubation. The main outcome measure was the peak PAED score in the recovery room after surgery. Delirium in the recovery period was defined as a PAED score of 10 or higher. There was pain when the pain scale was greater than 4 points [11]. The PAED score was only included in the analysis when the Richmond Manic Sedation Scale (Table 3) score was higher than − 2 and pain was unlikely to trigger manic behaviour [12]. If the excitement behaviour in the recovery room was improved after the administration of painkillers (fentanyl 1 µg/kg), the excitement behaviour during those periods was not ED. If a member of the study team was unable to obtain a PAED score in the recovery room, the child was excluded from further evaluation.

**Table 1:** Trial schedule of enrolment, interventions, and assessments

Time point	Study period					
	Enrolment	Allocation	Post-allocation			Closed-out
	− 1 D	0	Pre	After extubation 15 min	After extubation 15–30 min	After extubation 30 min
Enrolment						
Eligibility screen	X					
Informed consent	X					
Allocation		X				
Assessments						
PAB score			X			
FLACC score				X	X	X
RAS score				X	X	X
PAED score				X	X	X

**Table 2:** General condition of the patients

**Patient characteristics**	**All patients** (***n*)**	**Burst suppression was present (*n*)**	**P 值**
Age			
Height			
Weight			
ASA score			
1			
2			
3			
Male *n* (%)			
Premature delivery *n* (%)

**Table 3:** Intraoperative variables

		**All patients** (***n*)**	**BS** (***n*)**	***P *值**
Type of surgery *n* (%)	Orthopaedics			
	Urology department			
	Thoracic surgery			
	Burn and plastic surgery department			
	General surgery			
Anaesthesia induction modality	Intravenous induction			
	Inhalation induction			
Intubation modality	Laryngeal mask placement			
	Endotracheal intubation			
Mode of anaesthesia maintenance	Intravenous anaesthesia			
	Inhalational anaesthesia			
Neuromuscular blockade	Yes/no			

#### Secondary observation indicators

1. Determine the time factors related to burst suppression, including induction, sevoflurane inhalation concentration (3%), the number and total amount of propofol intravenous injection, intubation/laryngeal mask, regional block anaesthesia, skin incision, end of skin suture, awakening, extubation, and other periods.
2. Determine the participant factors related to burst suppression, including age, weight, sex, premature birth history, ASA grade, and previous anaesthesia history
3. Determine the perioperative factors related to burst suppression, including:
  A. Anaesthesia factors: anaesthesia mode, medication, anaesthesia duration, etc.
  B. Operative factors: operation type, location, blood transfusion, infusion, etc.
  C. Physiological factors: anaesthesia induction status (Table 4, Paediatric Anaesthesia Behaviour scores, PAB), blood pressure, heart rate, oxygen saturation, body temperature, electrolytes, etc.
  D. Resuscitation: extubation time, eye opening time, delirium during awakening, PACU time, and recovery quality

### Project risk pre-assessment and risk disposal plan

This test method was used to carry out routine operations on the participants. It did not involve drug use, retention of body fluid samples, and other intervention behaviours, let alone invasive intervention such as retention of tissue samples. Therefore, it did not cause significant adverse reactions or harm to the participants. If adverse events did occur, they were recorded, handled, and reported in a timely manner.

Delirium during the awakening period was mostly a self-limiting process. If delirium occurred during the awakening period, the observation time in the recovery room was extended until delirium disappeared. If necessary, medication was given or consultation with the neurology department was requested.

### Statistical treatment

Within the outcome variables, categorical variables include emergence delirium and burst suppression, continuous variables include awakening time, extubation time, PACU time, and recovery quality, etc. Data were statistically analysed using the SPSS 23 statistical software. Histograms and the Kolmogorov–Smirnov test were employed to assess normality. Continuous variables are presented as mean ± standard deviation or median (interquartile range). To assess differences between the two groups, *t*-tests were used for normally distributed continuous variables, and the Mann–Whitney *U* or Wilcoxon rank sum tests were used for non-parametric distributed continuous variables. The chi-square test will be used for categorical variable analysis (or Fisher’s exact test if cell counts are < 5. The Kruskal–Wallis test was used for multiple comparisons, and Bonferroni correction was applied for post hoc comparisons. Statistical significance was set at *P* < 0.05.

#### Baseline data

Baseline and general data are presented by standard statistical methods, continuous variables are normally distributed using means, SDS/non-normally distributed measurements data are presented using medians (interquartile ranges), and categorical variables such as sex are presented using percentages.

#### Main outcome measure analysis

The analysis of the main indicators included all participants who met the inclusion and exclusion criteria and completed the study. The main measures were analysed using descriptive statistics and logistic regression, respectively:

1. Number of burst suppressions and duration of each event
2. Burst suppression was expressed as a percentage of the total time in each period
3. To explore the relationship between burst suppression and wake-up delirium, we used chi-square or Fisher’s exact tests with the outcome of wake-up delirium as a dichotomous and dependent variable to analyse the relationship between the relevant categorical variables and dichotomous dependent variables. Our primary analyses were used to create the multivariate logistic regression models to assess the association between emergence delirium and perioperative variables. Odds ratios (ORs) with 95% confidence intervals (CIs) for each factor were calculated in the logistic regression. Variables with statistically significant values (*P* < 0.1) in the univariate model were entered into the multivariate model. Predictors tested included age, sex, body mass index (BMI), the PAB score, type of surgery, duration of fasting water, and anaesthesia maintenance mode. Model diagnostics were also reported, including the Hosmer–Lemeshow goodness-of-fit test, a receiver operating characteristic (ROC) curve, and the area under the curve (C-index). Only variables significant at the *p*-value 0.05 level were retained.

## Results

A report of the current research findings is provided in Tables 2, 3, and 4.

**Table 4:** EEG and physiological data at various time periods

	**Induction**	**Pre-incision**	**Surgical**	**Postsurgical**	**Total**
Burst suppression events					
Prevalence *n* (%)					
Total number					
Total duration					
Average duration					
Percentage of burst suppression EEG/total time (median [IQR])					
SpO_2_ (%)					
SBP (mmHg)					
MAP (mmHg)					
Heart rate (beats per minute)					
Temperature (Celsius)					
EtCO2 (mmHg)					
Airway resistance (cmH_2_O)					

## Discussion

ED in childhood is a common neurological complication, mainly characterised by confusion, loss of directional force, no eye contact, and restlessness. In addition, it can increase the risk of self-injury, dehiscence of the surgical wound, and occurrence of postoperative adverse behaviours such as intravenous catheter displacement and can eventually be a threat to patients, parents, and primary caregivers [13]. Burst suppression is the alternation of electrical activity between isoelectric and brief bursts, often secondary to various factors, such as infantile encephalopathy, deep anaesthesia, hypoxic-ischaemic trauma, coma, and hypothermia [14]. To explore the factors associated with wake-up delirium and perioperative EEG, in a previous study, it was reported [15] that epileptiform discharges during the induction of anaesthesia are associated with wake-up delirium, which suggests that we can reduce the occurrence of epileptiform discharges by EEG monitoring during induction and further reduce the incidence of wake-up delirium. In addition, the type of inhalational anaesthetic has some influence on the occurrence of paediatric wake-up delirium. Rapid awakening was a risk factor for delirium during the awakening period. Sevoflurane and desflurane awakenings are rapid, and postoperative delirium is more likely to occur with these anaesthetics than with other inhalational anaesthetics. Intravenous anaesthetics, such as propofol, have a well-established sedative versus hypnotic effect, and studies have found that continuous intraoperative pump or surgical bi-bolus administration of propofol reduces the incidence of wake-up delirium and is one of the most desirable drugs to prevent and treat ED in children. Similarly, the type of surgery also has an impact on ED in children. A prospective cohort study [16] demonstrated that the incidence of wake-up delirium in children is > 25% in both ophthalmological and otolaryngological surgery, > 15% in both urological and general surgery, and only 6% in other surgeries. Premedication may also be effective in preventing ED, and a meta study reported [17] that premedication with midazolam and clonidine was effective in reducing the incidence of ED during general anaesthesia surgery if the agents were maintained with sevoflurane. No premedication was used in this trial with the aim of excluding the influence of confounding factors on the outcome of this experiment.

The evaluation criteria of wake-up delirium are not uniform at present, and the PAED scale is the most widely used assessment method at this stage, but there are many subjective factors, as well as some errors in the judgement of experimental results that need to be further refined in the future. The primary objective of this ongoing prospective, observational study was to determine the association between EEG burst suppression under general anaesthesia in children and several adverse outcomes, including postoperative awakening delirium as well as prolonged awakening, and to reduce the incidence of awakening delirium while ensuring appropriate depth of anaesthesia and safety during general anaesthesia in children. We aim to provide additional reference value for paediatric anaesthesiologists in predicting the occurrence of paediatric wake-up delirium.

## Trial status

This study is currently at the patient enrolment and data collection stage and was approved by the Institutional Review Board of Beijing Children’s Hospital, Capital Medical University on December 13, 2021 (iec-c-006-a04-v.06). It was registered in Chinese clinical trial registry on January 5, 2022 (ChiCTR2200055256). The current version of the study protocol is version 1.1. The first participant will be recruited on January 6, 2022, and is expected to be finished by 31 January 2023. The conclusions of this study will be published in a peer-reviewed journal.

## Data Availability

The datasets supporting the conclusions of this article are included within the article (and its additional files).

## Abbreviations

ED: Emergence delirium
BS: Burst suppression
EEG: Electroencephalogram
PAED: Paediatric Anaesthesia Emergence Delirium
PACU: Anaesthesia recovery room
TIVA: Total intravenous anaesthesia
ASA: American Association of Anesthesia
FLACC: Face, Legs, Activity, Cry, and Consistency
PAB: Paediatric Anaesthesia Behaviour

## References

[1] Fritz BA, Kalarickal PL, Maybrier HR (2016). Intraoperative electroencephalogram suppression predicts postoperative delirium. Anesth Analg.

[2] Fritz BA, Maybrier HR, Avidan MS (2018). Intraoperative electroencephalogram suppression at lower volatile anaesthetic concentrations predicts postoperative delirium occurring in the intensive care unit. Br J Anaesth.

[3] Pedemonte JC, Plummer GS, Chamadia S (2020). Electroencephalogram burst-suppression during cardiopulmonary bypass in elderly patients mediates postoperative delirium. Anesthesiology.

[4] Akeju O, Westover MB, Pavone KJ (2014). Effects of sevoflurane and propofol on frontal electroencephalogram power and coherence. Anesthesiology.

[5] Akeju O, Pavone KJ, Westover MB, et al. A comparison of propofol- and dexmedetomidine-induced electroencephalogram dynamics using spectral and coherence analysis [published correction appears in Anesthesiology. 2015 Apr;122(4):958. Lei, Gao [corrected to Gao, Lei]]. Anesthesiology. 2014;121(5):978–989. 10.1097/ALN.0000000000000419. 10.1097/ALN.0000000000000419 PMC4304638 25187999

[6] Chandler JR, Myers D, Mehta D (2013). Emergence delirium in children: a randomized trial to compare total intravenous anesthesia with propofol and remifentanil to inhalational sevoflurane anesthesia. Paediatr Anaesth.

[7] Inouye SK, Westendorp RG, Saczynski JS (2014). Delirium in elderly people. Lancet.

[8] Akeju O, Pavone KJ, Thum JA (2015). Age-dependency of sevoflurane-induced electroencephalogram dynamics in children. Br J Anaesth.

[9] Cornelissen L, Kim SE, Purdon PL, Brown EN, Berde CB (2015). Age-dependent electroencephalogram (EEG) patterns during sevoflurane general anesthesia in infants. Elife.

[10] Kanaya A (2016). Emergence agitation in children: risk factors, prevention, and treatment. J Anesth.

[11] Shi M, Miao S, Gu T, Wang D, Zhang H, Liu J (2019). Dexmedetomidine for the prevention of emergence delirium and postoperative behavioral changes in pediatric patients with sevoflurane anesthesia: a double-blind, randomized trial. Drug Des Devel Ther.

[12] Koch S, Stegherr AM, Rupp L (2019). Emergence delirium in children is not related to intraoperative burst suppression - prospective, observational electrography study. BMC Anesthesiol.

[13] Mohkamkar M, Farhoudi F, Alam-Sahebpour A, Mousavi SA, Khani S, Shahmohammadi S (2014). Postanesthetic emergence agitation in pediatric patients under general anesthesia. Iran J Pediatr.

[14] Chalia M, Lee CW, Dempsey LA (2016). Errata: Hemodynamic response to burst-suppressed and discontinuous electroencephalography activity in infants with hypoxic ischemic encephalopathy. Neurophotonics.

[15] Koch S, Rupp L, Prager C (2018). Emergence delirium in children is related to epileptiform discharges during anaesthesia induction: an observational study. Eur J Anaesthesiol.

[16] Voepel-Lewis T, Malviya S, Tait AR (2003). A prospective cohort study of emergence agitation in the pediatric postanesthesia care unit. Anesth Analg.

[17] Zhang C, Li J, Zhao D, Wang Y (2013). Prophylactic midazolam and clonidine for emergence from agitation in children after emergence from sevoflurane anesthesia: a meta-analysis. Clin Ther.

